# Radiological Findings of Woodhouse-Sakati Syndrome: Cases Reported From Saudi Arabia

**DOI:** 10.7759/cureus.28540

**Published:** 2022-08-29

**Authors:** Arwa M Alzahrani, Lamis O Alsuwailem, Rinad M Alghoraiby, Fahad B Albadr, Yahya M Alaseri

**Affiliations:** 1 Department of Radiology and Medical Imaging, King Saud University Medical City/College of Medicine, King Saud University, Riyadh, SAU; 2 Department of Radiology, King Abdulaziz Medical City, Riyadh, SAU

**Keywords:** mri, genetics, neurological disorders, neuroradiology, woodhouse-sakati syndrome

## Abstract

Woodhouse-Sakati syndrome (WSS) is a rare autosomal recessive neurodegenerative genetic disorder caused by mutations in the DCAF17 gene. It primarily manifests with endocrinological symptoms such as hypogonadism, failure to develop secondary sexual characteristics, diabetes, and hypotrichosis. Neurological manifestations include intellectual disabilities, dystonia, dysarthria, and hearing loss.

This paper describes the cases of two Saudi Arabian sisters, aged 37 and 36, who were born to first-degree consanguineous parents. They had normal growth and development except for certain intellectual disabilities. However, they were presented with primary amenorrhea and no secondary sexual characteristics at puberty, and they were subsequently diagnosed with WSS. The first patient presented with dysmorphic features, dysarthria, tremors, and dystonia. The second patient presented with hypotrichosis, predominantly affecting the temporo-occipital regions, and cerebellar signs on physical exam. Both patients had hair thinning and bilateral sensorineural hearing loss. Brain MRI of both patients showed increased iron deposition in the basal ganglia and multiple faint T2-FLAIR (fluid-attenuated inversion recovery) hyperintensity foci involving the centrum semiovale, corona radiata, and peritrigonal white matter bilaterally. MRI abdomen of the second patient revealed early hepatic fibrosis, with diffuse moderate to severe hepatic steatosis reaching a fat fraction of 19%, and increased intensity of the splenic vein with multiple collaterals. Further research is needed to achieve a better understanding of this syndrome to improve patient care and outcomes.

## Introduction

Woodhouse-Sakati syndrome (WSS) is a rare neurodegenerative genetic disorder with an autosomal recessive mode of inheritance caused by mutations in the DCAF17 gene. It is a multi-systemic syndrome that primarily affects the endocrine and nervous systems in the context of increased iron deposition in the brain [1].

Most cases reported were from the Middle East, likely related to the consequences of consanguinity. Phenotypic variability of this syndrome, even among siblings, has been observed, adding to the challenges of acquiring a correct diagnosis promptly [2].

The objective of this report is to describe the clinical features of two Saudi Arabian sisters, diagnosed with WSS, who were born to first-degree consanguineous parents, to better characterize the variable presentations of this syndrome.

## Case presentation

Patient 1

Clinical Findings

The first patient is 36 years old. Her early life passed without major medical problems apart from some intellectual difficulties. The patient presented with primary amenorrhea, bilateral hearing loss, hair thinning, and later developed dysarthria. On physical examination, the patient was found to have dysmorphic features as well as oral, facial, and cervical dystonia, tremors in both hands, and a dystonic gait.

Diagnostic Assessment

The whole exome sequencing test showed homozygous variant c.436del p. (Ala147 Hists*9) in the DCAF17 gene. The laboratory findings revealed prediabetes and hypothyroidism (Table 1).

**Table 1:** Laboratory findings

Labs	Level	Reference Range
Hemoglobin A1C (HgA1C)	6.2%	4–5.6%
Thyroid Stimulating Hormone (TSH)	7.3 mlU/L	0.25–5.0 mIU/L
Free T4 (FT4)	4.8 pmol/L	11.4–22.7 pmol/L

**Figure 1:**
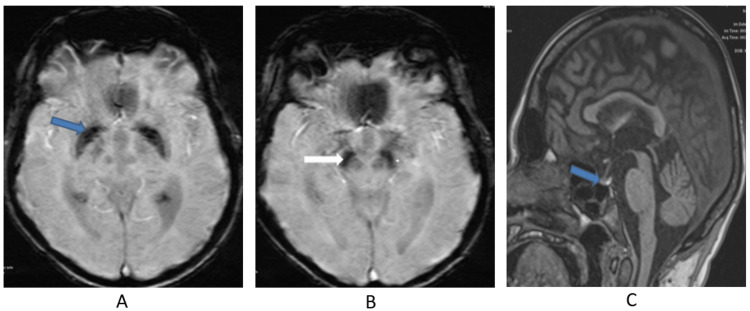
Images A and B are axial susceptibility weighted imaging (SWI) of the brain showing putaminal blooming artifacts reflecting iron accumulation and iron deposition in the substantia nigra (blue and white arrows). Image C is a sagittal T1-weighted MRI showing a partially empty sella and a small pituitary gland (blue arrow).

Upon imaging, the patient’s brain MRI showed iron deposition in multifocal areas, including the substantia nigra (Figures 1A, 1B), and an empty sella turcica with pituitary hypoplasia (Figure 1C).

Patient 2

Clinical Findings

The second patient, a 37-year-old female, had an uneventful childhood except for some intellectual difficulties. The patient did not develop any secondary sexual features or have her menarche when she reached the age of puberty. She presented to the gynecological clinic at the age of 29 with primary amenorrhea; by the time she presented, she had previously been on hormonal replacement therapy (HRT) for ten years, which had failed to induce menarche. Hair loss, dizziness, progressive hearing loss, and tinnitus were all symptoms reported by the patient.

Her physical examination found that she lacked secondary sexual characteristics and had diffuse hair loss. Her neurological evaluation revealed bilateral moderate to severe sensorineural hearing loss, overshooting saccades, a slight overshoot in the finger-to-nose test, and impaired tandem gait.

Diagnostic Assessment

**Figure 2:**
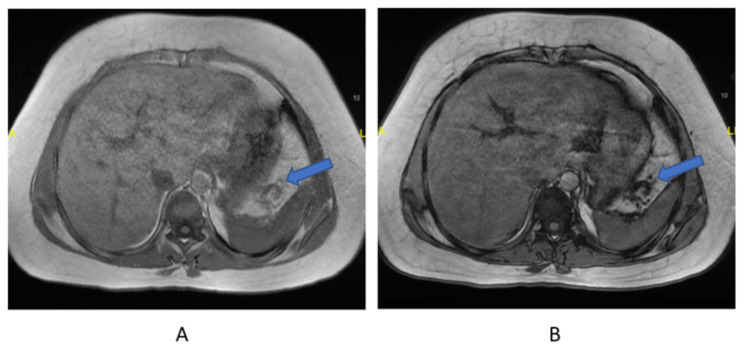
Images A and B are axial inphase and outphase Lava Flex MRI of the abdomen showing early hepatic fibrosis with diffuse moderate to severe hepatic steatosis and increased intensity of the splenic vein with multiple collaterals likely to be chronically thrombosed (blue arrows).

**Figure 3:**
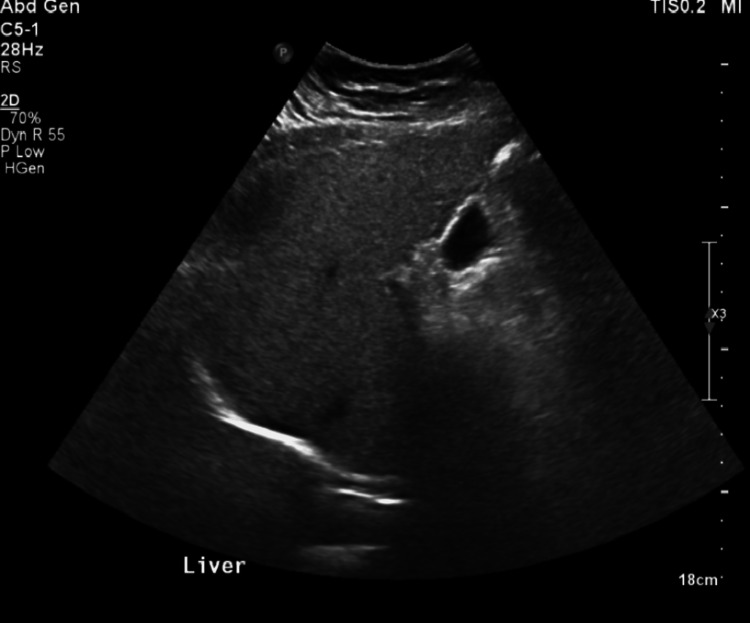
An abdominal ultrasound (US) showing mild diffuse fatty infiltration and a slight coarse echo pattern.

Investigations similar to the first patient were carried out and she was subsequently diagnosed with WSS. Whole exome sequencing revealed a homozygous variant c.436del p (Ala147 Hists*9) in the DCAF17 gene. Her laboratory investigations also revealed elevated ferritin levels, raising the possibility of hemochromatosis. And after further investigations, an MRI of the abdomen was performed to reveal early hepatic fibrosis with diffuse moderate to severe hepatic steatosis reaching a fat fraction of 19%, and increased intensity of the splenic vein with multiple collaterals likely to be chronically thrombosed (Figures 2A, 2B). An abdominal ultrasound (US) was also performed, which revealed mild diffuse fatty infiltration as well as a slight coarse echo pattern (Figure 3). Following the US, other possible causes of liver cirrhosis, including viral and autoimmune, were ruled out by performing hepatitis B serology, antinuclear antibodies, and anti-smooth muscle antibodies.

**Figure 4:**
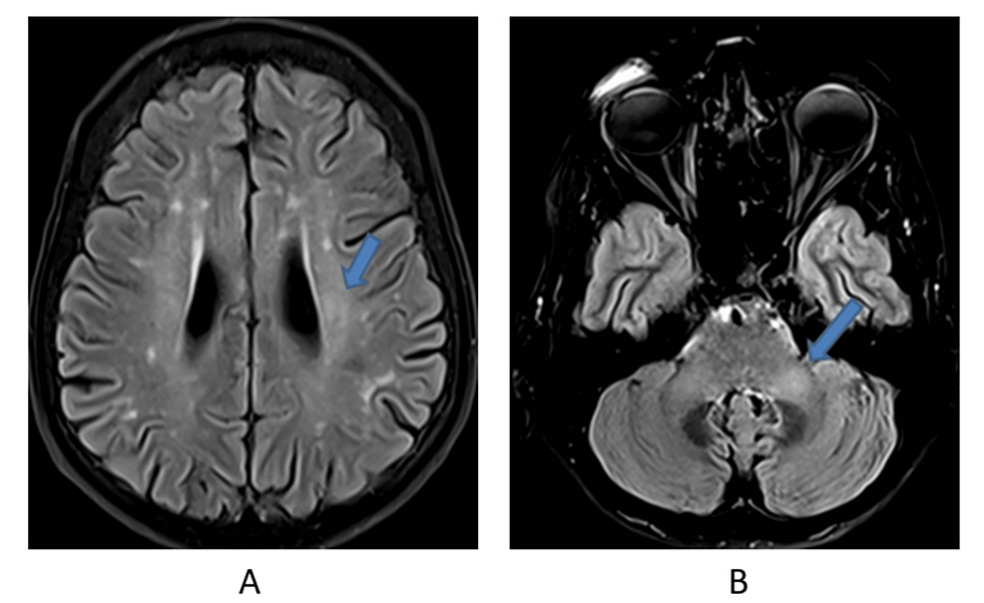
Image A is an axial FLAIR MRI of the brain showing a mild degree of white matter lesions, with faint periventricular hyperintensity (blue arrow). Image B is an Axial FLAIR MRI of the brain showing a mild degree of white matter lesions, with faint bilateral middle cerebellar peduncle hyperintensity (blue arrow). FLAIR: fluid-attenuated inversion recovery

The patient underwent a brain MRI investigation which revealed multiple periventricular and cerebellar hyperintense lesions (Figures 4A, 4B).

Therapeutic Interventions

Both patients were managed symptomatically with a multidisciplinary approach that included gynecology to monitor HRT with oral contraceptives, dermatology to treat alopecia with minoxidil 2% as well as ear nose, and throat (ENT), and audiology collaboration to employ hearing aids and speech therapy. The first patient is being managed at the endocrinology clinic for hypothyroidism via thyroxine and diabetes mellitus with lifestyle management. For dystonia, antispasmodic agents include baclofen and trihexyphenidyl for controlling extrapyramidal symptoms. The second patient who was found to have liver cirrhosis is being further investigated for fatty liver disease and an early picture of liver cirrhosis and will be then managed accordingly. Both patients have regular follow-ups at the aforementioned clinics.

## Discussion

WSS is a rare autosomal recessive genetic disorder manifesting mainly with neurological and endocrinological abnormalities that was first described in 1983 in various Saudi Arabian consanguineous families [3]. The prevalence of WSS is unknown. However, to date, 180 cases from more than 85 families have been reported in the literature [2].

WSS mainly presents with hypogonadism, failure to develop secondary sexual characteristics, diabetes mellitus, and hair thinning progressing to alopecia. Neurological symptoms include intellectual disability of varying severity, sensorineural hearing loss, dysarthria, and dystonia. People who possess this syndrome have characteristic dysmorphic features such as hypertelorism, triangular face, and prominent nasal bridge. Rarely, cardiac and ophthalmological symptoms may be present [2,4-6]. In a review article of 58 Qatari cases diagnosed with WSS, it was found that all had alopecia as well as hypogonadism, and only 46% had diabetes mellitus [2]. In contrast, a systematic review by Agopiantz et al. found that all 72 patients diagnosed with WSS developed hypogonadism, alopecia, and decreased insulin growth factor-1 (IGF-1) levels, the latter possibly playing a role in the development of diabetes mellitus in this disorder [7]. 

This report revealed the cases of two female siblings that demonstrated the typical picture of WSS which is consistent with the ‘triad’ reported by Agopiantz et al [7]; they were born to first-degree consanguineous parents who were both healthy, proving the autosomal recessive mode of inheritance of this syndrome. The onset of their clinical presentation was comparable to prior studies [2]. For instance, they both experienced a relatively uneventful early life with normal development and growth, except for a mild form of mental disability. Primary amenorrhea and progressive alopecia predominantly affecting the occipital and temporal region during adolescence and early adulthood were the trigger for a series of investigations that ultimately led to the confirmation of both sisters' diagnoses of WSS. Both siblings demonstrated the progressive nature of this syndrome as they gradually exhibited a variety of symptoms associated with WSS, including sensorineural hearing loss, extrapyramidal movement, and hyperglycemia. In the second case, liver cirrhosis -- a unique finding not previously documented in the literature -- was found. It is still unclear, though, whether or not this discovery is directly connected to WSS.

In addition to the possibility of intestinal anomalies in WSS [5], a more recent study reported the presence of urogenital anomalies in the form of uterine and ovarian agenesis, possibly contributing to hypogonadism in such cases [8]. Our study, however, did not perform a pelvic MRI in order to rule out this possibility and instead concluded that a small hypophysis of the pituitary was likely the cause of hypogonadism [1].

On brain MRI, the most common findings in individuals with WSS are prominent basal ganglia iron deposits, small pituitary glands, and progressive white matter lesions predominantly affecting the frontoparietal and periventricular regions, which were all findings reported in both our cases; a characteristic sparing of the subcortical U-fibers was also noted. Less frequently observed findings include prominent perivascular gaps and constrained diffusion in the corpus callosum splenium, which were not present in both our cases. Of note, the clinical presentation of dystonia in the absence of radiological evidence of iron deposition in the brain was reported by Abusrair et al [1]. Although brain MRI findings suggestive of WSS are of aid when there is a high clinical suspicion of the syndrome, a normal brain MRI does not exclude the disease [2].

There is no standard treatment for WSS. Instead, supportive treatment and regular follow-ups are the current standards of care for the management of this disease as the exact pathophysiology is still ambiguous. Hypogonadism is managed with life-long HRT to induce menarche and develop secondary sexual characteristics. The main mechanism for diabetes in WSS is thought to be caused by β-cell function defects, prompting us to think that insulin might play a role in treatment. Hearing aids can be used for hearing loss and speech therapy can aid in dysarthria. For extrapyramidal symptoms, oral medication can be tried, however, botulinum toxin injections are usually preferred. Hypothyroidism is managed with the standard treatment, and alopecia is mainly treated with minoxidil [4,7,9].

Limitations of this study include lack of IGF-1, luteinizing hormone (LH), and follicular stimulating hormone (FSH) measurement for further classification of hypogonadism, and inaccessibility of gynecological imaging.

## Conclusions

WSS is an uncommon genetic systemic disease with widely variable presentations, mainly affecting the neuroendocrine system. Neuroradiological findings of this rare syndrome include a hypoplastic pituitary gland, pronounced iron accumulation in the basal ganglia, and white matter lesions which are predominantly found in the frontoparietal and periventricular regions. The pathogenesis of WSS remains to be not fully understood and the treatment is symptomatic.

To the best of our knowledge, this is the first case of asymptomatic liver cirrhosis, as evident radiologically, in the setting of WSS. Further research is needed to establish a causal relationship between liver cirrhosis and WSS, and to achieve a better understanding of this syndrome to aid in achieving a prompt diagnosis, early treatment, and familial genetic counseling. By establishing this, there may be an improvement in patient care and quality of life. This is of great importance as most of the signs and symptoms of WSS appear after childhood.
